# Cropland expansion in Ecuador between 2000 and 2016

**DOI:** 10.1371/journal.pone.0291753

**Published:** 2023-09-19

**Authors:** José I. Ochoa-Brito, Aniruddha Ghosh, Robert J. Hijmans

**Affiliations:** 1 Department of Environmental Science and Policy, University of California, Davis, Davis, California, United States of America; 2 Spatial Informatics Group, LLC, Pleasanton, California, United States of America; 3 International Center for Tropical Agriculture (CIAT), Nairobi, Kenya; K University of Fisheries and Ocean Studies, INDIA

## Abstract

We describe changes in the cropland distribution for physiographic and bioregions of continental Ecuador between 2000 and 2016 using Landsat satellite data and government statistics. The cloudy conditions in Ecuador are a major constraint to satellite data analysis. We developed a two-stage cloud filtering algorithm to create cloud-free multi-temporal Landsat composites that were used in a Random Forest model to identify cropland. The overall accuracy of the model was 78% for the Coast region, 86% for the Andes, and 98% for the Amazon region. Cropland density was highest in the coastal lowlands and in the Andes between 2500 and 4400 m. During this period, cropland expansion was most pronounced in the Páramo, Chocó Tropical Rainforests, and Western Montane bioregions. There was no cropland expansion detected in the Eastern Foothill forests bioregion. The satellite data analysis further showed a small contraction of cropland (4%) in the Coast physiographic region, and cropland expansion in the Andes region (15%), especially above 3500m, and in the Amazon region (57%) between 2000 and 2016. The government data showed a similar contraction for the Coast (7%) but, in contrast with the satellite data, they showed a large agricultural contraction in the Andes (39%) and Amazon (50%). While the satellite data may be better at estimating relative change (trends), the government data may provide more accurate absolute numbers in some regions, especially the Amazon because separating pasture and tree crops from forest with satellite data is challenging. These discrepancies illustrate the need for careful evaluation and comparison of data from different sources when analyzing land use change.

## Introduction

Croplands cover around 112% of the global non-frozen land surface, and another 22% of land is used for grazing [1, 2]. Cropland areas are expanding in response to the continuing increase in the global demand for food and other agricultural products due to population growth, the development of new agricultural products such as biofuel, and changes in diets [3–5]. This expansion threatens biodiversity and the provisioning of ecosystem services [6–10]. Furthermore, climate change may facilitate the expansion of agriculture into areas at higher latitudes and elevations [6]. Thus, to understand the impact of agriculture on the environment it is important to monitor on-going land use change.

Government statistics can be used to analyze patterns of agriculture expansion. However, these are not always available, or not frequently updated, and sometimes unreliable [11]. Moreover, these records tend to be aggregated to regional and national levels that can preclude precise understanding of the importance of the changes that are occurring. The availability of multi-decadal remote sensing data has created an alternative data source for monitoring cropland dynamics [12]. However, remote sensing-based estimates of land use could also be unreliable due to a number of factors, including persistent cloud cover, and spectral similarity between land cover classes (e.g. forest and tree crops). Remote sensing applications to monitor agricultural expansion tend to either focus on large regions, such as continents or the whole world, or on specific areas where the process is related to rapid changes such as deforestation [13] such as in the Brazilian Amazon [14]. To better understand agricultural land use, more detailed studies of other regions are needed as well.

Here we describe changes in the distribution of crop production in continental Ecuador. Recent agricultural expansion has been reported from some regions in Ecuador [15, 16] but there is no comprehensive study of recent changes in cropland distribution for the entire country. We use Landsat data to map cropland distribution in continental Ecuador for the years 2000 and 2016. We use the results to describe changes in cropland distribution by physiographic region, bioregion, elevation and longitude. We found substantial discrepancies between the Landsat based results and government statistics and we discuss possible reasons for these differences.

## Methods

### Study area

**Fig 1:**
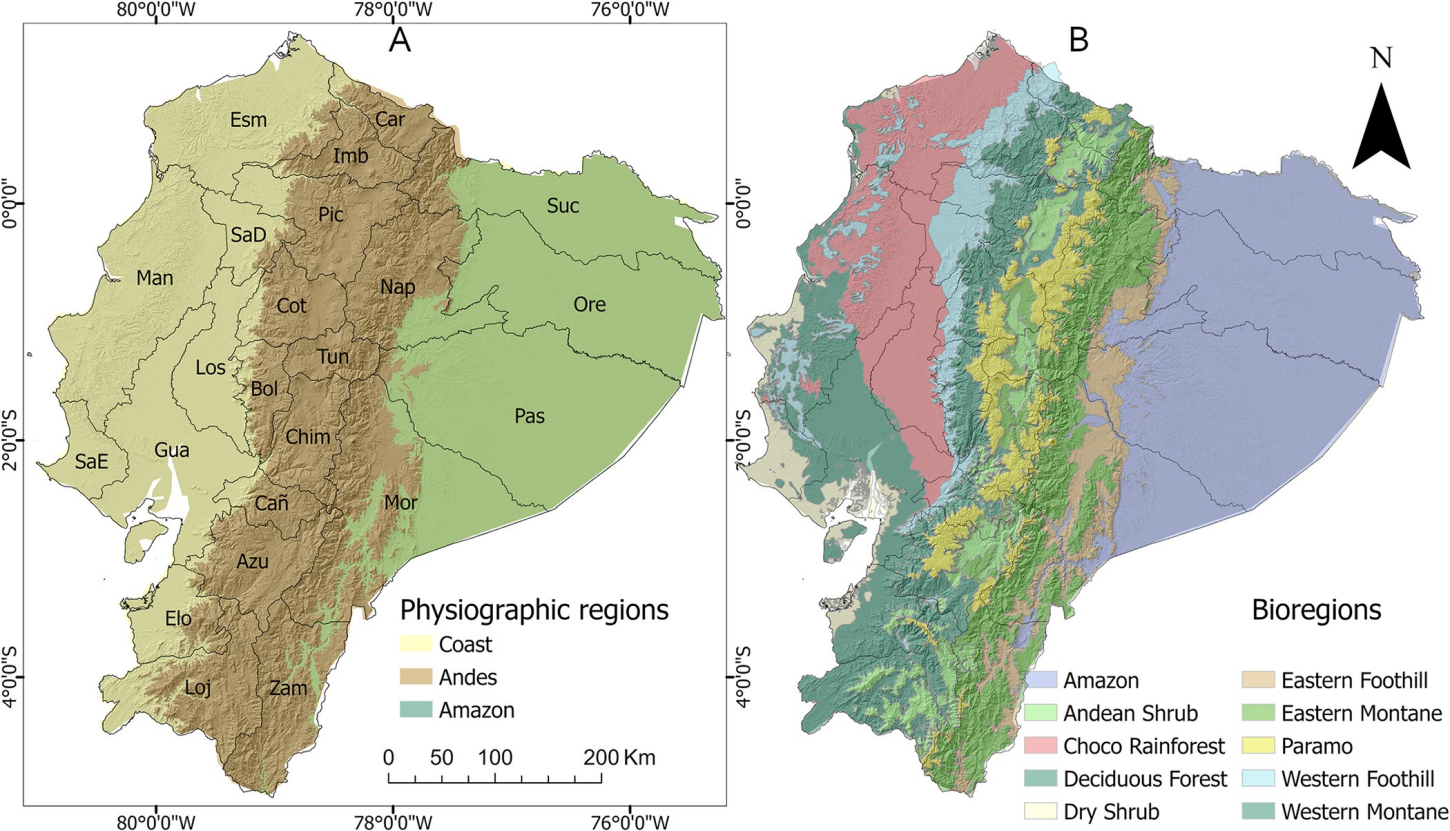
Continental Ecuador. (A) The three major physiographic regions and province boundaries. (B) Bioregions and province boundaries (after Ron [20]; and Sierra et al. [21]). Province codes: Esm = Esmeraldas, Man = Manabí, SaE = Santa Elena, Gua = Guayas, Los = Los Ríos, Elo = El Oro, Car = Carchi, Imb = Imbabura, Pic = Pichincha, SaD = Santo Domingo de los Tsáchilas, Cot = Cotopaxi, Bol = Bolívar, Tun = Tungurahua, Chim = Chimborazo, Car = Cañar, Azu = Azuay, Loj = Loja, Suc = Sucumbíos, Ore = Orellana, Pas = Pastaza, Mor = Morona Santiago, Zam = Zamora Chinchipe.

We studied cropland expansion in continental Ecuador (that is, excluding the Galapagos Islands) (Fig 1). The study area has 23 provinces (first level administrative subdivisions) across three physiographic regions: Coast, Andes and Amazon (Fig 1A), and 10 bioregions (Fig 1B). The Amazon consists of a single bioregion of tropical lowlands naturally covered with rainforest. The Coast also has lowlands, that are very humid regions in the north (Choco bioregion), but less moist in the south (Dry Shrub bioregion). The Andes (also referred to as Sierra), has elevations between 1000 and 6260 m above sea level with a variety of climates and vegetation types. The Ecuadorian Andes are relatively humid in comparison to the Andes further south in Peru and Bolivia. The natural vegetation between the tree-line (~ 3250 m) and the perpetual snow line is dominated by a grassland ecosystem called páramo [17] that has a high biodiversity of plants [18] and serves as water source for lower regions due to its higher precipitation and deep soils.

Coastal agriculture is dominated by large-scale production of export crops such as banana, coffee, cacao, and sugar cane. Andean farmers mostly produce food crops such as maize, potato, cereals, and vegetables. Agriculture in the Amazonian lowlands mainly consists of large plantations of oil palm, fruit trees and of cultivated pastures for raising livestock (13% of the Amazonian lowlands has cultivated pastures) [19].

We chose the years 2000 and 2016 to study land cover change because of the availability of Ecuadorian government statistics for land use, agriculture and forests, and because we did the land cover/land use data collection in 2016. In addition, Landsat 7 data has been available since April 1999 and provides complementary data for Landsat 8 catalog.

### Landsat data

We used Landsat data because of its availability for our study period, high spatial resolution of 30m, and adequate combined-revisit cycle. We used the surface reflectance (SR) collection 1 tier 1 time series from three Landsat satellites: Landsat 5 (Jan 01, 1984 –May 5, 2012), Landsat 7 (Jan 01, 1999 –present), both atmospherically corrected with the Landsat Ecosystem Disturbance Adaptive Processing System (LEDAPS) algorithm [22, 23], and Landsat 8 (Apr 11, 2013 –present) atmospherically corrected using the Land Surface Reflectance Code (LaSRC) (version 1.4.1) algorithm [24], to create composite images for the years 2000 and 2016. Atmospherically corrected SR products might enhance cropland detection in cloudy and foggy conditions by removing atmospheric noises. Although there are minor differences in the bandwidths between the satellite sensors (Table 1), several studies have shown that these variations are not significant when merging multi-temporal scenes for land cover classification purpose [25–34]. For example Zhu & Woodcock 2014 [35] used all the available Landsat data until 2014 including Landsat 4, 5, and 7, to generate the Continuous Change Detection and Classification (CCDC) algorithm to enhance land cover change detection.

**Table 1:** Wavelength (μm) of Landsat bands for the three Landsat sensors used in this study.

Landsat	Blue	Green	Red	NIR	SWIR I	SWIR II	Thermal
5 TM	0.45–0.52	0.52–0.60	0.63–0.69	0.76–0.90	1.55–1.75	2.08–2.35	10.4–12.5
7 ETM+	0.45–0.52	0.52–0.60	0.63–0.69	0.77–0.90	1.55–1.75	2.09–2.35	10.4–12.5
8 OLI	0.45–0.51	0.53–0.59	0.64–0.67	0.85–0.88	1.57–1.65	2.11–2.29	10.6–11.19

### Cloud removal

Much of Ecuador remains cloudy throughout the year, especially the humid tropical lowlands, and this is major obstacle to satellite-borne optical remote sensing. To tackle this challenge, we tested multiple cloud removal techniques. First, we applied a two-stage cloud-screening to Landsat scenes for both 2000 and 2016. All Landsat SR products contain a pixel quality assessment (QA) band that has been generated by the CFmask algorithm [36]. Using the QA band we masked cloud and cloud shadow pixels. However, the remaining and permanent cloudy areas in combination with the low availability of Landsat scenes (especially for the year 2000), required a second cloud-filter. Some methods might not result effective in removing cirrus clouds in the dense cloud cover of the Ecuadorian tropics [37]. Other robust cloud-cover removal algorithms such as the automated cloud-cover assessment (ACCSA) [38] identify outliers corresponding to permanent cloudy locations. We implemented a similar approach by generating time series of Landsat TM SR in 2000 to evaluate the spectral patterns of sensitive bands to atmospheric haze (Blue) and cloud brightness (NIR) [39, 40] for a representative cloudy region. For this purpose, we selected a location within the persistent cloudy zone and traced a 0.25 km^2^ homogenous land cover polygon. The land homogeneity was visually evaluated in Google Earth with high spatial resolution historical images. These SR time series curves allowed us to identify and remove saturated dates (SR>1) corresponding to fog, cloud borders, and thin semi-transparent clouds not detected by the first filter [25, 41]. Although this significantly improved the composite quality for the year 2000, bright areas persisted due to local atmospheric conditions or sensor radiometric limitation, perhaps because the ETM+ spectral channels cannot easily detect semitransparent clouds such as Cirrus Uncinus and Fibratus, and cloud edges [42]. We also evaluated a reflectance percentile filter over the SR collections at 10, 20, 50% to remove residual cloud and cloud-shadow.

### Gap-filling and compositing

The failure of the Landsat 7 ETM+ scan-line corrector (SLC) in May 2003 produced data with constant wedge-shaped gaps between scans [43]. We created gap-filled ETM+ scenes using data from other dates [44]. Finally, to create a cloud free mosaic, we used a five-year window of cloud-masked images for 2000 (January 1998 to December 2002) and 2016 (January 2014 to December 2018). Merging contemporaneous Landsat sensors, L5+L7 for 2000, and L7+L8 for 2016, increased revisit frequency until eight day and helped to supersede mosaic mismatching, missing tiles, and invariable cloudy locations corresponding to thin clouds that were not removed by the second cloud filter. We produced the final cloud-free composite based on median reflectance value for each pixel for the visible (blue, green, red), near infrared, shortwave infrared (one and two), and thermal bands.

### Classification model

**Fig 2:**
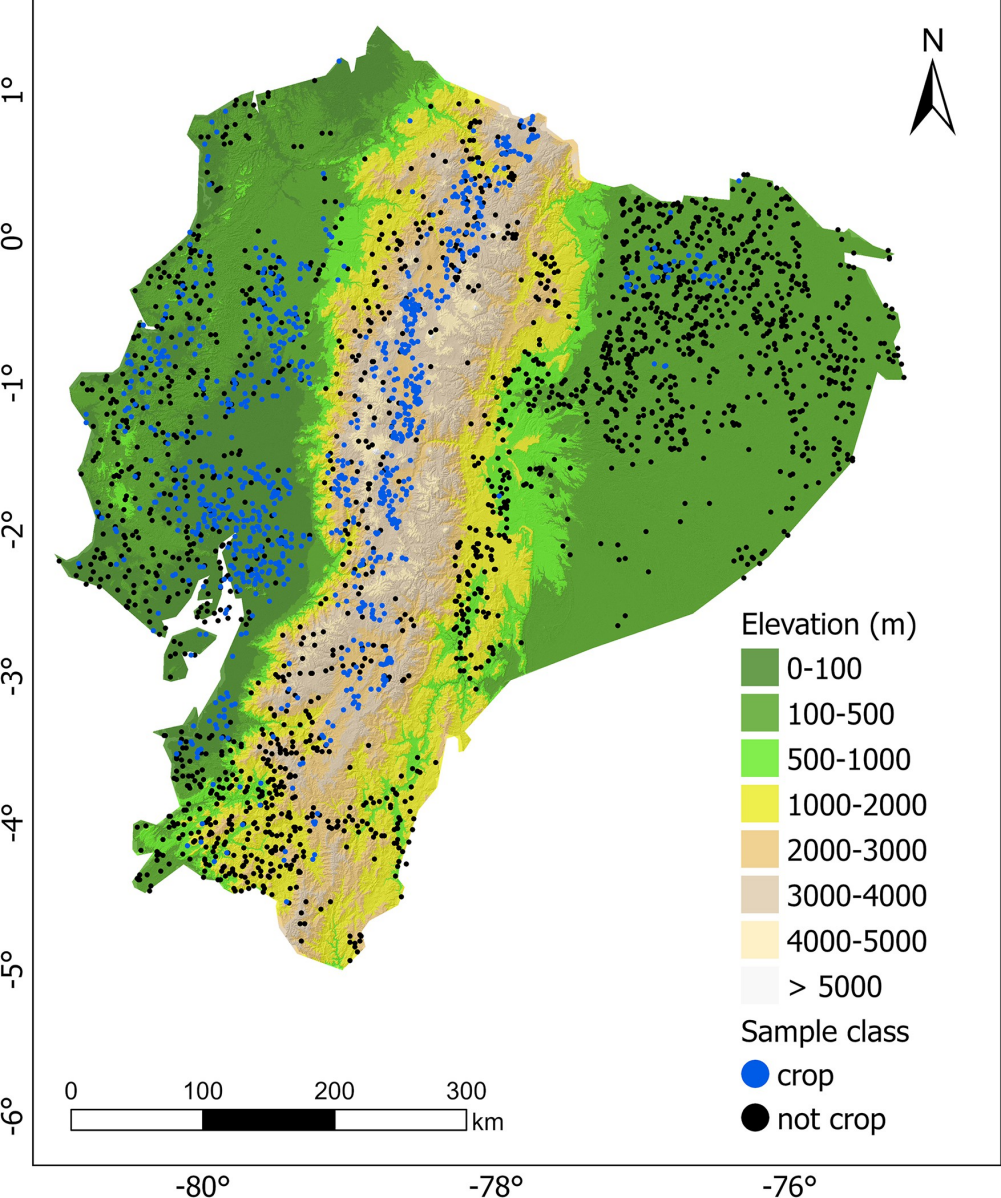
Elevation in Ecuador and the spatial distribution of sample locations that were classified as “crop” or “not crop” and used to train the Random Forest model. There are fewer samples in areas with persistent cloud cover.

We collected a total of 3010 land cover samples (34% crop, 66% no-crop) by visual interpretation of high-spatial resolution satellite imagery (~1.85m or higher) using the open-access Geosurvey platform (https://geosurvey.qed.ai/about/). The locations were generated randomly over the study area (Fig 2). Sample locations that were much affected by clouds, or were very heterogeneous, were not used. The sample locations were filtered over available cloud-free scenes for 2016 and were sampled using a 250 × 250 m window. We labeled annual and perennial crops, cultivated pastures, and fallow areas as cropland. Fallow areas are land that is clearly used for crop production, but at the time of sampling did not have a crop on it. No-crop class consisted of all other vegetated non-crop and non-green areas. Due to the unavailability of high-resolution images, training samples from 2000 were not collected, instead we used the model trained with data for 2016 to classify the corresponding 2000 dataset.

We used Random Forest (RF) to classify pixels as cropland and non-croplands [45, 46]. RF is a machine learning algorithm widely accepted for satellite-image classification purposes. RF utilize a set of multiple decision trees to choose the most popular class providing an advantage over other tree-based classifiers by selecting random subsets of features and minimizing correlation among the decision trees [47]. The model was trained separately by physiographic region (Coast, Andes, Amazon) and for the entire country with a parametrization of 100 trees, 10 predictors, and 3 out of 10 randomly selected variables per tree. These parameters were determined to be optimal with cross-validation. The predictor variables included were seven Landsat bands (Table 1), Normalized Difference Vegetation Index (NDVI) (Eq 1), Enhanced Vegetation Index (EVI) (Eq 2), and 30-meter elevation from the Shuttle Radar Topography Mission (SRTM) [48]. NDVI is an indicator of greenness and biomass for vegetation monitoring [49] while EVI offers improvement in sensitivity over dense vegetation cover minimizing canopy soil variation. Elevation is strongly associated with climate and cropping practices in Ecuador [50].


$$NDVI=\frac{NIR-RED}{NIR+RED}$$ (Eq 1)

$$EVI=2.5\frac{NIR-RED}{NIR+6*RED-7.5*BLUE+1}$$ (Eq 2)

In the equations above, NIR is the reflection in the near-infrared part of the spectrum, RED in the red part, and BLUE in the blue part of the spectrum.

**Fig 3:**
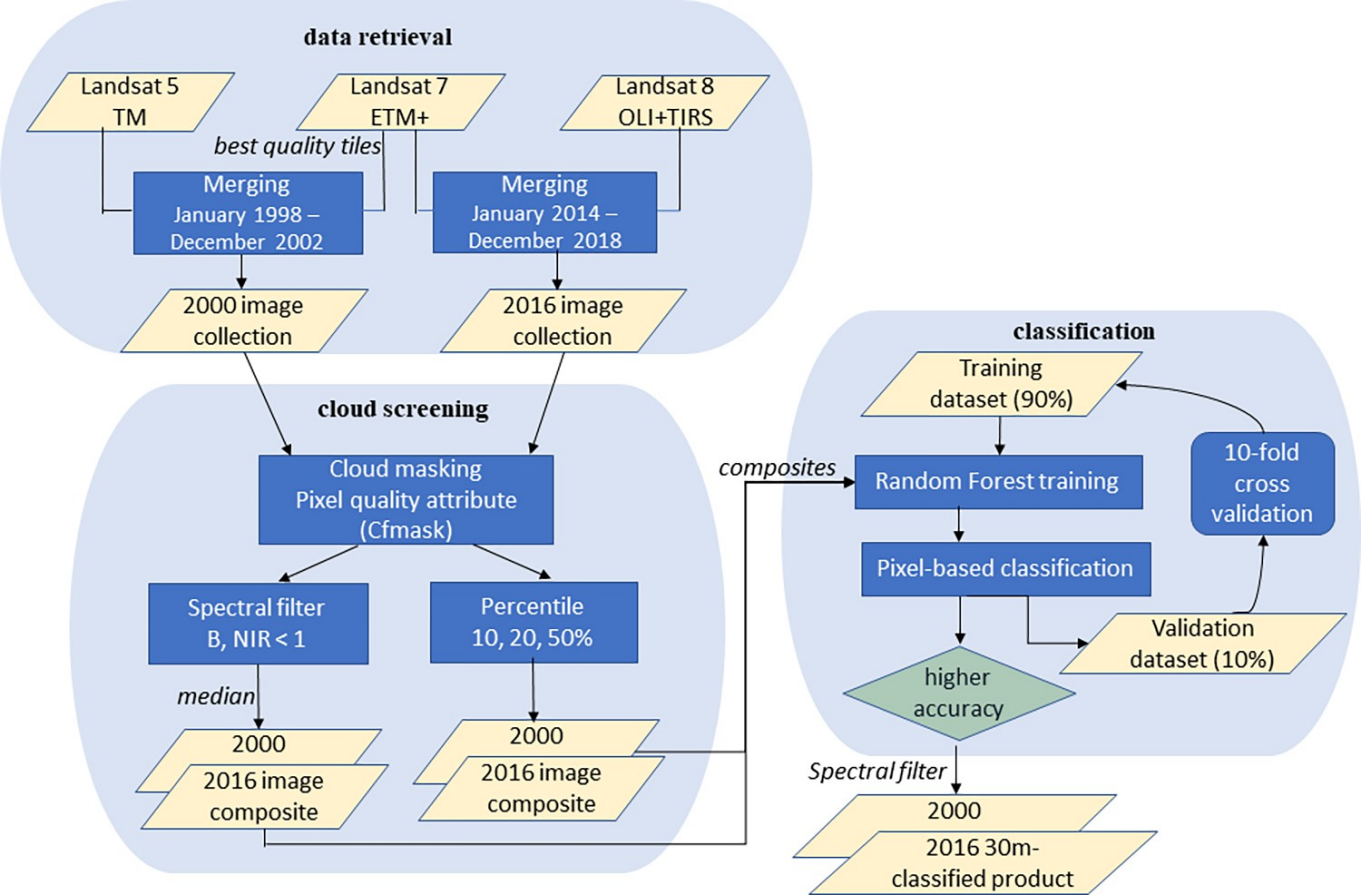
Workflow of the methodology for our satellite-based classification.

For the year 2016, we evaluated model accuracy for the physiographic regions and at entire study region using 10-fold cross-validation (CV) by computing the overall accuracy (OA), kappa coefficient, producer accuracy (PA), and user accuracy (UA) [8]. The kappa coefficient quantifies the agreement between two variables chance on a [0, 1] scale. A high kappa indicates that the agreement is not likely to be due to chance alone [51]. PA measures the error of omission for a class, while the UA indicates the error of commission. The entire modeling workflow is presented in Fig 3.

### Comparison with census and survey data

**Fig 4:**
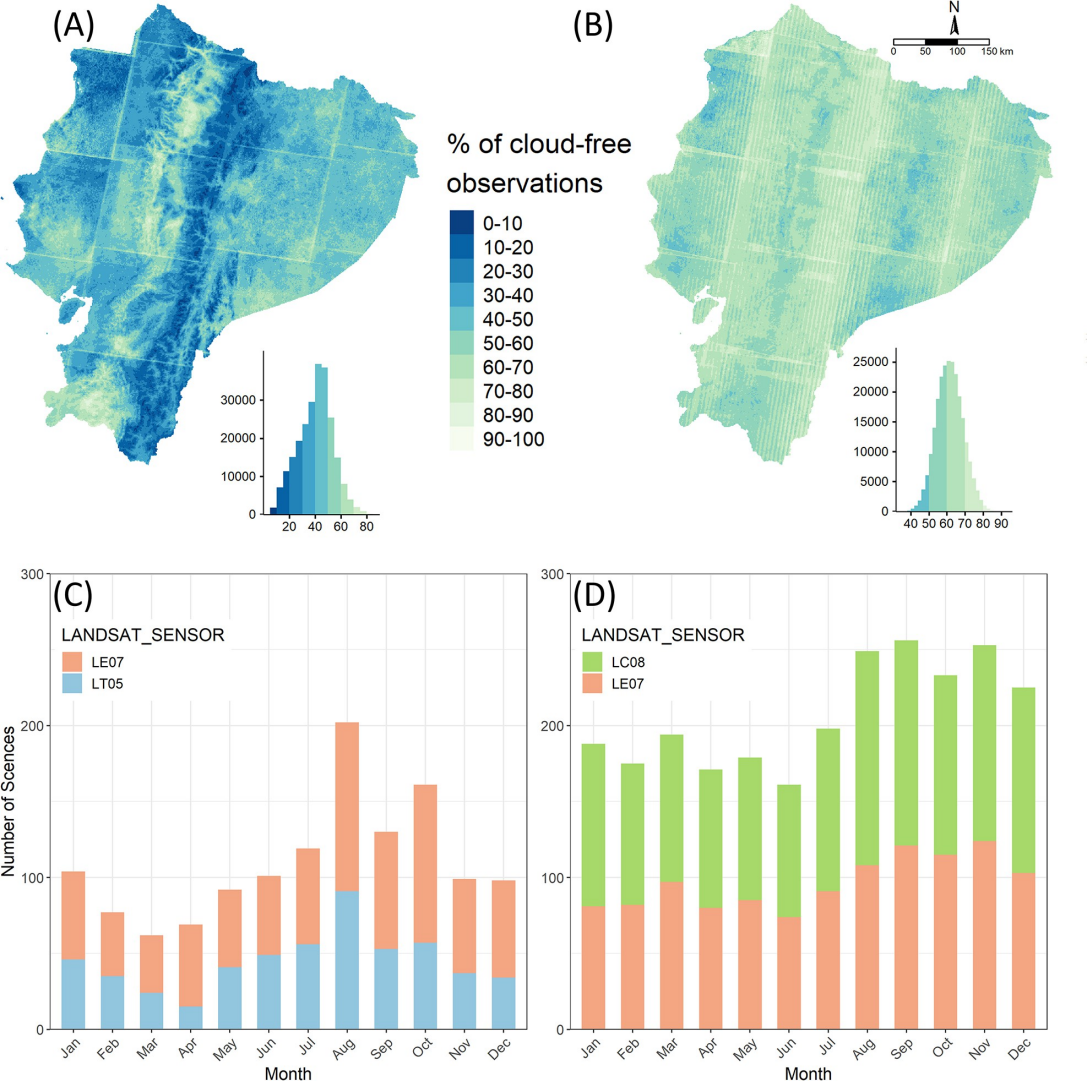
Landsat data availability in a 5-year period. (A) Pixel-wise fraction of cloud free observations for January 1998 –December 2002 composting period (2000); (B) Pixel-wise fraction of cloud free observations for January 2014 –December 2018 composting period (2016). (C) Monthly number of cloud-free observations available for 2000. (D) Monthly number of cloud-free scenes available for 2016.

We compared our model estimated cropland areas with government statistics at national and province level for both time periods. We compiled two crop distribution data sets provided by the Ecuadorian government: the census for year 2000 and survey data for year 2016. These data are made available by the National Institute of Statistics and Census of Ecuador (INEC) through his project of Agricultural Area and Production Survey or “*Encuesta de Superficie y Producción Agropecuaria Continua (ESPAC)”* [19]. These data were generated using a methodology that follows the Food and Agriculture Organization of the United Nations (FAO) standards for land use data collection. ESPAC considers annual and perennial crops, fallowed land, and cultivated pastures within the cropland category. Both datasets provide statistics of cropland area by agricultural land use type and by province. The 2000 census has data for 21 provinces, while the 2016 survey data has data for 23, including for the two more recently formed provinces Santa Elena and Santo Domingo de los Tsáchilas (Fig 4). We summarized the data by administrative region: Coast, Sierra, Amazon, and calculated proportions of disagreement with our remote sensing estimations. The Coast comprises six provinces, the Sierra is formed by the 11 central provinces, and the Amazon region has six provinces. We included cultivated pastures in cropland, but we excluded natural pastures, as was done for the high-resolution image training sample collection.

### Geographic distribution of cropland across elevation and longitude

We used our Landsat based estimates of cropland extent to describe some main geographical patterns of crop distribution. We computed the fraction of cropland area by 50 m elevation bands, and by 0.1° longitude intervals. We also summarized the results by physiographic region and by ecoregion. We computed the fraction cropland for square raster cells with a 1 km^2^ spatial resolution to map agricultural extent in both years and to measure change. The satellite imagery retrieval, filtering, cloud-masking, and compositing were performed in Google Earth Engine (GEE) platform [52]; the land cover classification and subsequent data analysis were done with R version 3.6.2 [53] and the randomForest [54] and raster [55] packages.

## Results

### Cloud screening

For each pixel, were computed the percentage of observations (dates) available that were cloud-free in each time frame. This quantity differed strongly by bioregion due to satellite data availability and variability in weather conditions. For instance, the Eastern Andes and the Northern coastal lowlands showed particularly low rates of cloud-free observations in 2000 (0–30%) (Landsat 5 and 7) (Fig 4A). The proportion of clear-sky observations was significantly higher and more spatially homogenous in 2016 (Landsat 7 and 8) (Fig 4B).

**Fig 5:**
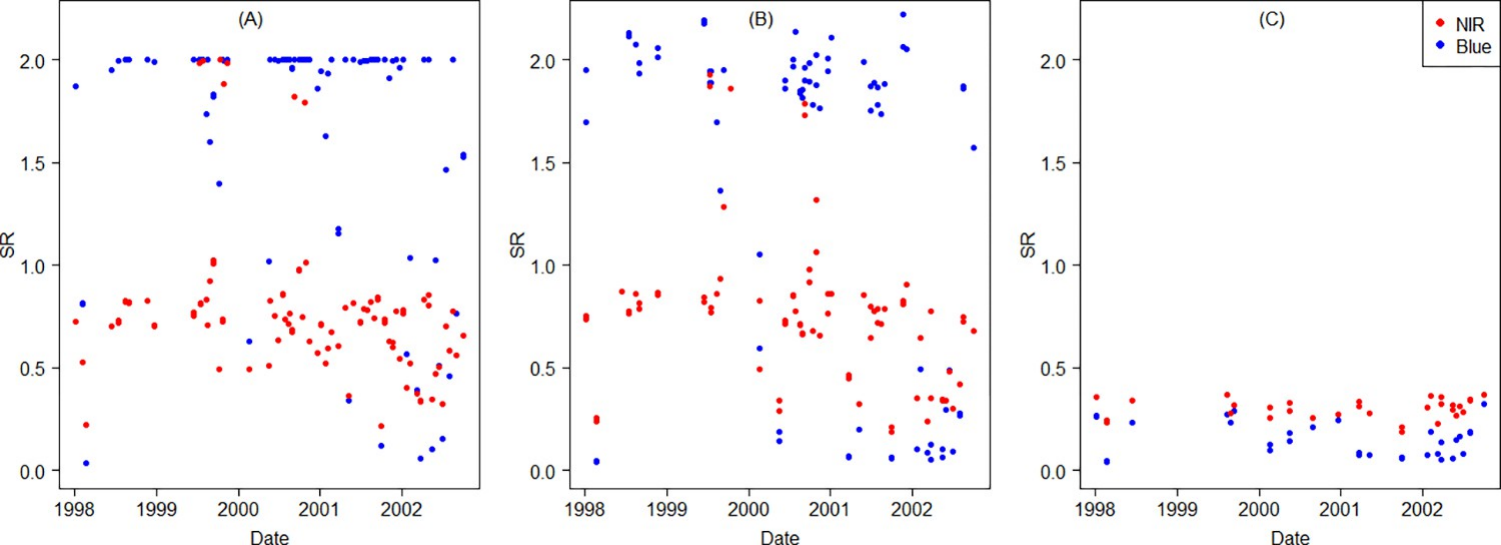
Time series of spatially averaged surface reflectance (SR) for a permanent cloudy location west of Quito in a five-year frame (1998–2002). Displaying SR for the Blue-NIR space, with the NIR displayed in red: (a) SR Composite; (b) Cloud-masked composite; (c) Cloud-mask plus spectral filter.

While Landsat 5 and 7 delivered from 60 (wet season) up to 200 (dry season) cloud-free observations combined across the study area in 2000 (Fig 4C), Landsat 8 played a fundamental role by significantly increasing the number of observations available in 2016 for combined compositing through a more stable and continual data delivery (Fig 4D). In addition, the number of Landsat 7 collected scenes by geographical location in 2000 is lower than the expected value from the revisit cycle due to temporal discontinuities of the Landsat archive on the acquisition plan [56]. The spectral cloud filter evaluated times series of surface reflectance (SR) (Fig 5). Saturated and very high SR values greater than 1 (SR>1) correspond to cloudy pixels (Fig 5A). After traditional cloud-masking was applied, several outliers remained visible corresponding to thinner cloudiness (Fig 5B). By adding spectral filtering (B, NIR < = 0.4), these persistent saturated values were removed (Fig 5C).

**Fig 6:**
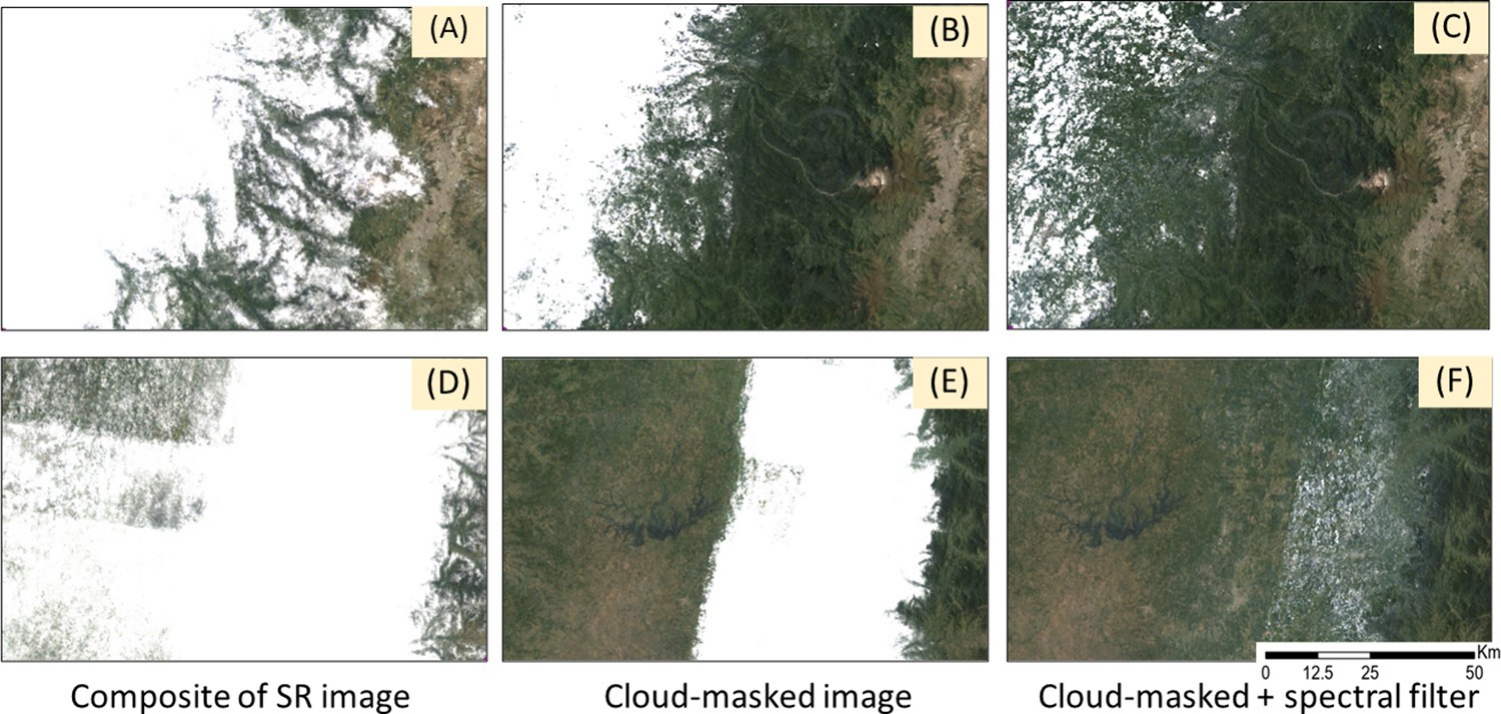
True color composites for different cloud removal approaches: none (A, D), cloud-masking (B, E), and cloud masking plus spectral filtering (C, F), for two permanent cloudy regions in 2000: the Western Montane bioregion (A-C) west of Quito, and the Chocó and Western Foothills (D-F).

The improvement on composite quality by adding a second filter was evident for representative cloudy sites in 2000 (Fig 6A and 6D). A large portion of clouds was removed with traditional cloud masking (Fig 6B and 6E). Permanent thin cloudiness was mostly removed only with spectral screening (Fig 6C and 6F).

With the percentile method, lower percentiles (10) were effective in removing pixels covered by semi-transparent clouds. However, using this method yielded cloud-free pixels for only 80% of the total area in 2000. The spectral analysis filter performed better in terms of quantity and quality of masked pixels, as it produced a cloud-free cover of 98% for 2000 and 100% of the study area for 2016.

### Accuracy of cropland detection

A 50-tree random forest parametrization resulted in a classification accuracy of 0.76 for Coast, 0.82 for Sierra, and 0.97 for Amazon of overall accuracy (OA). A 100-tree set-up achieved slightly better results with 0.76, 0.83 and 0.98 OA respectively. Using more than 100 trees did not enhance model performance. The OA of the Landsat based cropland detection was highest for the Amazon region, and lowest for the Coast (Table 2). The Andes (kappa = 0.7) and Amazon (kappa = 0.6) showed substantial agreement, while the agreement for the Coast was moderate (kappa = 0.5). The estimated cropland extent was affected by the cloud filter used. Also, there were large differences between our Landsat-based estimates and the government statistics, irrespective of the cloud filter used (Table 2). For the Coast region we obtained a producer accuracy (PA) of 0.71 and user accuracy (UA) of 0.77 for the crop class, and PA = 0.97 and UA = 0.96 for non-crop, Sierra region PA = 0.82 and UA = 0.8 for crop, PA = 0.79 and UA = 0.81 for non-crop, and for the Amazon region with PA = 0.81, UA = 0.78 for crop and PA = 0.8, UA = 0.83 for non-crop. For both classes, the errors of omission (100%-PA) in the Amazon and Sierra regions were lower than in the Coast; probably due to a higher crop heterogeneity and pasture presence in these regions.

**Table 2:** Accuracy assessment of cropland classification for continental Ecuador. Overall accuracy and kappa were computed with a 2016 validation sample. Disagreement with government statistics was computed with 2016 survey and 2000 census data.

Region	Year	Complementary cloud filter	overall accuracy (%)	kappa [0, 1]	estimated crop area (km^2^)	government crop area (km^2^)	disagreement with gov. statistics (%)
Coast	2016	*percentile (10–50)*	78.3	0.5	23,234	29,892	-22
		*spectral (blue*, *NIR)*	77.6	0.5	24,318		-19
	2000	*spectral (blue*, *NIR)*	-	-	25,321	32,039	-21
Andes	2016	*percentile (10–50)*	84.4	0.7	18,206	11,918	+53
		*spectral (blue*, *NIR)*	86.1	0.7	21,516		+81
	2000	*spectral (blue*, *NIR)*	-	-	18,724	19,622	-5
Amazon	2016	*percentile (10–50)*	97.1	0.6	1,253	5,218	-66
		*spectral (blue*, *NIR)*	97.5	0.6	2,335		-55
	2000	*spectral (blue*, *NIR)*	-	-	1,490	10,420	-86
Country	2016	*percentile (10–50)*	83.2	0.7	42,693	47,028	-9
		*spectral (blue*, *NIR)*	84.3	0.7	48169		+2
	2000	*spectral (blue*, *NIR)*	-	-	45,537	62,080	-27

The cloud free area was larger with the spectral filter, but the type of cloud screening used had very little impact on classification accuracy. Our 2016 cropland extent estimates were lower than the government survey data for Coast (-19%) and Amazon (-55%) while higher for the Andes (+81%) (Table 2). For 2000, our estimates were lower than the government survey for Coast (-21%), Amazon (-86%) and Andes (5%). The average accuracy for the three models, one for each physiographic region, was higher than that of the single model for the entire country (Table 2).

Visual inspection suggested that our model could not thoroughly differentiate between cropland-fallow transition and non-forest classes such as bare soil and abandoned land. We made visual inspection comparing our 2016 classified maps with high resolution mosaics at several random locations, and we found that some fallowed parcels were misclassified as non-crop because the similarity with bare soil. Also, in the Amazon and Coast permanent crops such as oil palm and coffee (tree crops) were not always identified as crops because of their spectral similarity with forest. PA and UA estimates are showing higher values for crop rather than for non-crop class. This makes sense since the non-crop class includes a range of land cover types.

### Comparison of cropland distribution with government statistics

**Fig 7:**
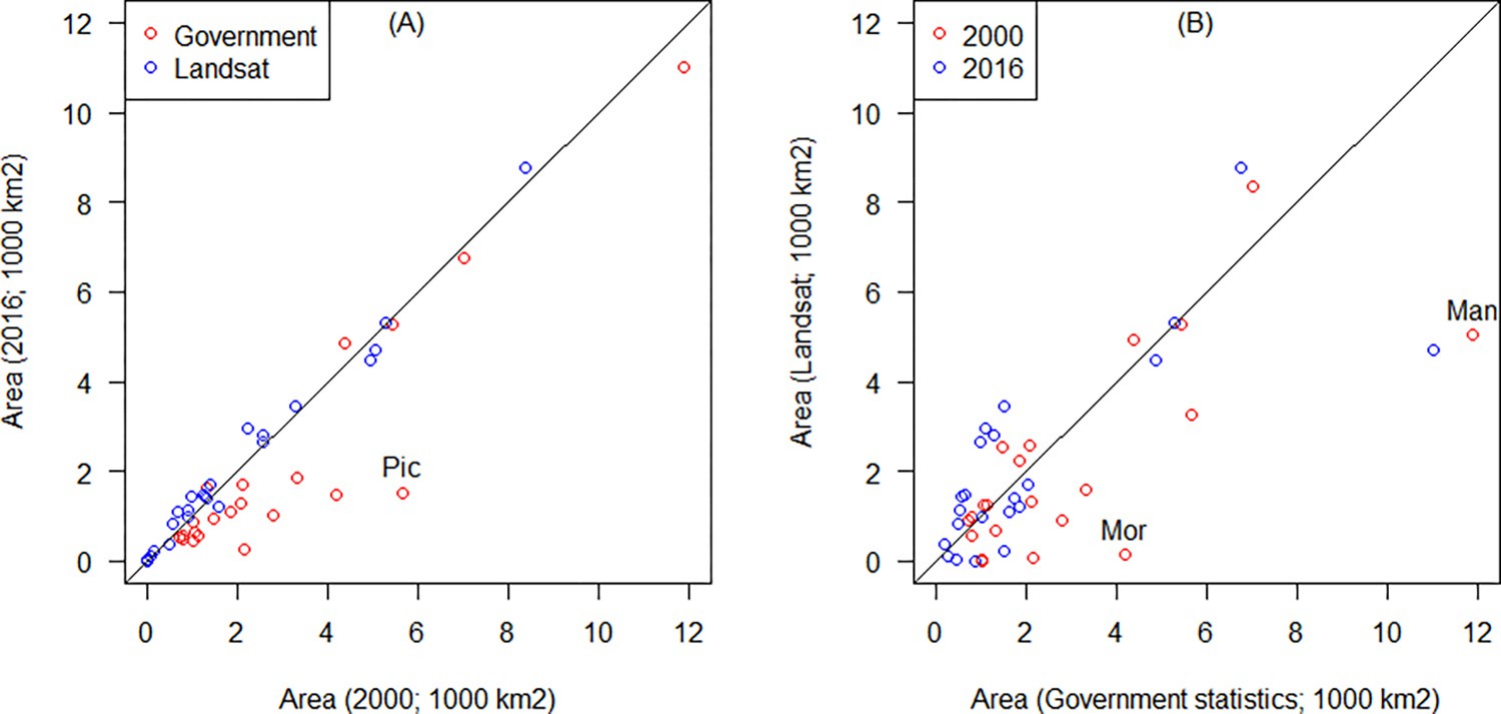
Comparison of cropland extent estimated by province of continental Ecuador. (A): cropland area for 2000 and 2016 according to the government statistics (2000 census and 2016 survey data); and Landsat estimates. (B): Government statistics versus our Landsat estimates for 2000 and for 2016. Province labels: Pic = Pichincha, Mor = Morona Santiago, Man = Manabí.

The satellite-based estimates had lower variation in province level crop area from 2000 to 2016 than the government data (Fig 7A). For example, the government statistics shows that the cropland area in Pichincha province (Pic) decrease with 73%, whereas our Landsat-based estimates suggest that it was 26%. There was high agreement for most of the provinces between the estimated and the government crop areas for 2000 and 2016 (Fig 7B), but with some notable exceptions for example, in 2000, the satellite-based estimate for Manabí province (Man) was 42% of the government provided number, while for Morona Santiago (Mor) this estimate was only 3% of the survey value. Both provinces are in tropical lowland areas.

Crop area by province and by physiographic region for 2000 and 2016 are provided in S1–S3 Tables.

### Spatial distribution of croplands

Our estimates of the presence of cropland in 2000 by physiographic regions were: Coast 32.9%, Andes 17.9%, and Amazon 0.6%. For 2016 our estimates were Coast 32.8%, Andes 21.1% and Amazon 1.3%. The *páramo* bioregion experienced the highest cropland expansion, from 13.3 to 21.4% (Table 3). Expansion in the Western Foothill forests and Andean Shrub bioregions was 2.8%. The Eastern Foothill Forests bioregion has least cropland (0%). By 2016 there was relatively little cropland in the Amazon (1.3%), Western Foothill (8.7%) and Eastern Montane forests (9.2%) compared to the other bioregions while >45% of the land in the Andean Shrub and Chocó Rainforest bioregions is used for crop production.

**Table 3:** The size of bioregions in Ecuador, and for 2000 and 2016, remote sensing based estimates of the percentage cropland, and the change in cropland area between these two years.

Physiographic region	Bioregion	Area (km^2^)	Cropland 2000 (%)	Cropland 2016 (%)	Change in cropland area (km^2^)
Amazon	Eastern Foothill	13,061	0	0	0
Amazon	Amazon	73,066	0.7	1.3	459.9
Andes	Western Montane	21,532	21.4	23.6	480.4
Andes	*Páramo*	15,906	13.3	21.4	1294.4
Andes	Andean Shrub	11,258	46.1	48.9	309.8
Andes	Eastern Montane	31,414	7.8	9.2	428.6
Coast	Dry Shrub	7,911	23.4	20.2	-249.9
Coast	Deciduous Forest	25,599	36.4	32.7	-952.1
Coast	Chocó Rainforest	31,587	45.3	47.5	693.2
Coast	Western Foothill	15,104	5.9	8.7	419.6

### Change in crop land distribution

**Fig 8:**
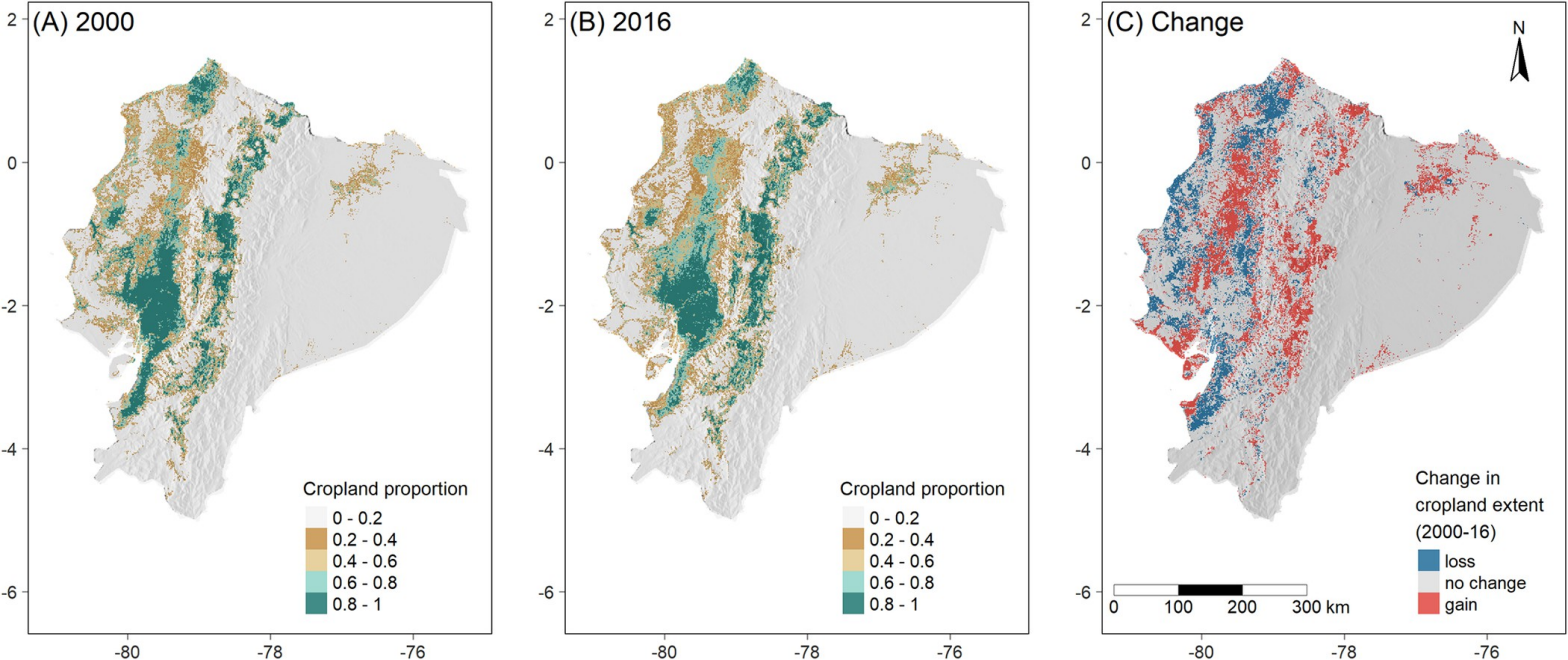
Cropland distribution in Ecuador in 2000 and 2016 based on classification of Landsat remote sensing data. Cropland area (proportion of land area) in (A) 2000; and (B) 2016; (C) change in cropland extent between 2000 and 2016.

At the national level, our land cover classification showed 45,536 km^2^ of cropland extent for 2000 (Fig 8A), and 48,169 km^2^ for 2016 (Fig 8B), suggesting an increase of 5.8% of agricultural land over this 17-year period (Fig 8C). At the physiographic region level, we found a slight reduction of in cropland area in the Coast (4%), whereas agricultural areas increased by 15% in the Andes and 57% in the Amazon.

We detected cropland expansion in the central-northern part of the Coast region, and much cropland loss along the coast (Fig 8C). In contrast, in the Andes and Amazon we mostly found agricultural expansion. Most of the expansion we detected was in central-eastern Andes, especially in Chimborazo province. In the Amazon, detected cropland increases were mostly in the area near the border of the northern provinces of Sucumbíos and Orellana.

### Analysis by elevation and longitude

**Fig 9:**
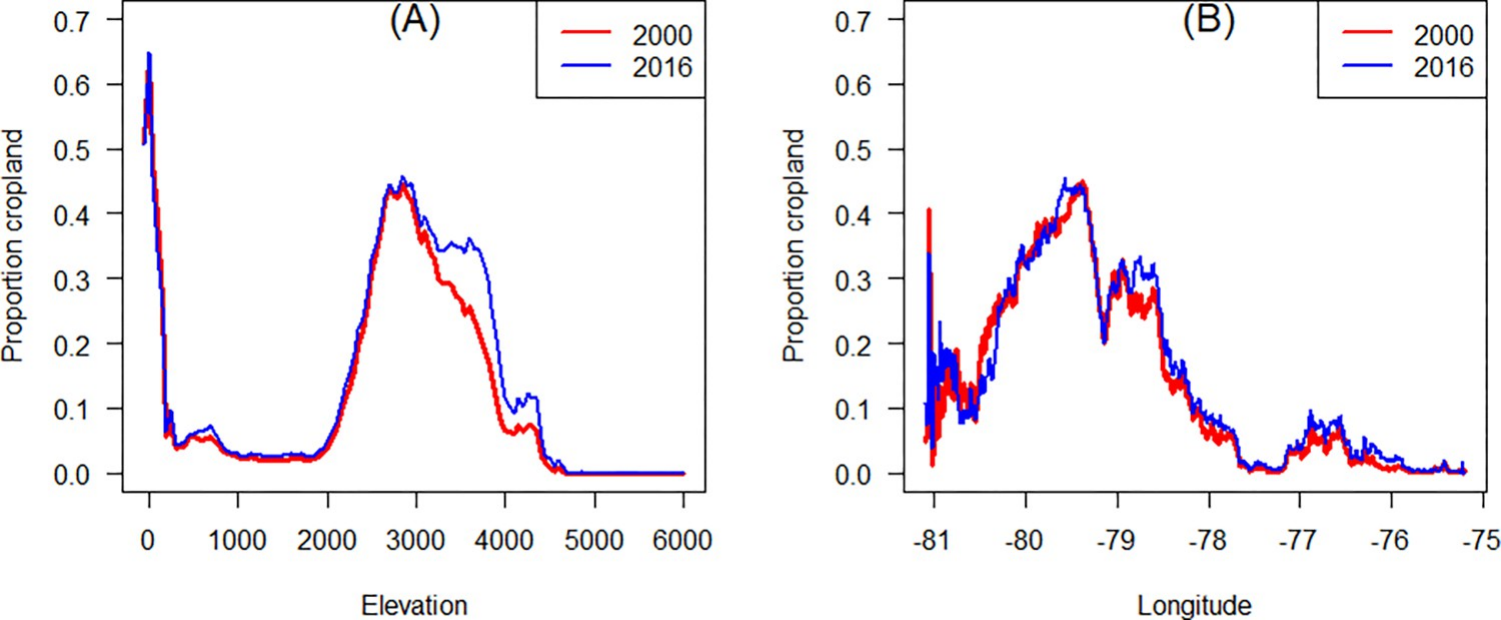
Geographical distribution of cropland in Ecuador by elevation (A) and longitude (B).

The proportion of land used for crops was highest in the coastal lowlands, around 100 m and 200 m above sea level, and in the Andes, between 2000 and 4000 m. Crop density is much lower at intermediate elevations and in the Amazon lowlands west of 76° W (Fig 9A). Our estimates suggest that cropland expanded most at elevations between 2800 and 4400 m, with an expansion of about 10% around 3500 m. This is also notable in Fig 5B that shows in increase between 78.5–79° W, as well as some expansion in the Amazon (between 76 and 77° W) (Fig 9B).

## Discussion

We used Landsat data to study cropland distribution in Ecuador between 2000 and 2016. We found major discrepancies with government reported data for the same time period. The government data showed a strong decline in the cropland area across the Andes and Amazon regions whereas our analysis suggest that cropland increased in the regions. A strong decline in cropland is not in line with our understanding of the study area and the literature. For example, Gray and Bilsborrow [57] found cropland expansion in the Andes and the Coast. Agricultural expansion in the Andean *páramo* bioregion was described by Ross et al. [58], who found 29% of cropland expansion between 3500m and 5000m elevation between 1999 and 2014 in the Río Chambo basin in the central Ecuadorian Andes. In addition, Wigmore and Gao [59] found a *páramo* cover loss of 22% between 1988 and 2007 in the Pambamarca area in northern Ecuador.

We did not find evidence of large-scale cropland abandonment that would support the scale of cropland loss reported by the government. The cropland expansion we detected in the northern Amazon around 76.5° W is also supported by Viteri-Salazar & Toledo, 2020 [60], who analyzed expansion of the agricultural frontier in the Ecuadorian Amazon and they estimated around 2190 km^2^ of cropland expansion between 2000 and 2011. While we show significant cropland increase in the Amazon, comparison with the government statistics and visual inspection suggested that we strongly underestimated the cropland area in that region. Even though we detected much more cropland in this region that other remote-sensing based efforts. For example, the Global Food Security-support Analysis Data at 30m (GFSAD30) Project for South America (GSFAD30SACE) [61, 62] detected 278 km^2^ of cropland for 2015, while our estimate was 2335 km^2^ for 2016. Further efforts to map croplands in rainforest areas could focus more on additional methods (e.g. pattern recognition or object-based image analysis) to distinguish between trees in a forest from trees in a plantation. Another challenge is to distinguish cropland from pastures exclusively used for grazing.

There is also a need for further development of complementary cloud-masking techniques such as spectral analysis of reflectance time series in tropical regions to address the problem of persistent and lower clouds. Synthetic aperture radar (SAR) may be an alternative data source for these areas with consistent cloud cover or foggy conditions [63, 64] By combining radar data with optical satellite observations we may improve land cover classification going forward for current and future time frames. However, the availability of SAR data continues to be a constraint for this approach, especially for both historical era as well as the current period after the Sentinel 1B failure that resulted in significant data gap. It could be of interest to study the importance of cross-sensor calibration for different classification use cases and how the results depend on the sensor characteristics, complexity of landscape, temporal duration of the composite etc., however this is beyond the scope of the current study.

Our results show that there can be large uncertainty in land cover data, whether from government statistics or from remote sensing data. Government agricultural statistics can be of very high quality if they are based on a census, and if the data are made available at a high spatial resolution. Such products can play a key role in calibrating satellite-based models that can be used for the years between censuses. Other survey methods can, in principle, also yield high quality data but our results suggest that, if the 2010 census is reliable, the 2016 survey underestimated agricultural land use in the Andes and Amazon regions. While the trend in cropland area in the Amazon suggested by the government data may not be reliable, the overall magnitude of the government estimates of cropland in that region may be more accurate than the remote-sensing based estimate. The census and the survey reported much more cropland in the Amazon than we, and other studies, detected with remote sensing and our visual inspection confirmed that we misclassified much land with pasture and tree crop, despite the very high accuracy that we computed (0.97 overall accuracy for the Amazon).

In satellite-derived products, data quality can be affected by cloudiness, which is a significant obstacle in the humid tropics. In this work, a minor percentage of pixels (~12%) from the 2000 satellite coverage mosaic remained cloud-masked corresponding to permanent cloudy locations and affected by Landsat data discontinuity and downlinking limitation on the Landsat 5 spacecraft [43]. Through visual inspection we noticed some non-filtered cloud pixels may have been misclassified as crops which might have led to certain overestimation of the 2000 cropland extent and crop contraction from 2000 to 2016.

We found cropland expansion in the Andes between 3000 and 4400 m between 2000 and 2016, mostly corresponding to the *páramo* bioregion, which provides various ecosystem services, for example, water supply for inter-Andean valleys [17, 18]. The transformation from natural *páramo* tussock grasses vegetation to cropland or to short grass vegetation by intensive grazing, expose the Andosol soils to direct sunlight, causing drying, water repellency, and significant reduction in soil water retention [65–67]. In the Coast, we observed a more stable cropland dynamics along the elevational and longitudinal gradient. However, significant agricultural expansion took place in the northern Coast at 79° W where there was a loss of lowland, montane and cloud-belt rainforests [68].

Future research could look at different crop types to better understand the reasons behind the differences between our remote sensing derived estimates and the government statistics. This further analysis could provide additional insights about deviations in estimated cropland extents across different provinces and bioregions in Ecuador due to differences between spectral signatures for individual crops and similarities with signatures for natural vegetation cover.

## Conclusion

Our study offers one of the first spatially detailed reports on cropland dynamics in Ecuador. We found 57% cropland expansion in the Amazon and 15% in the Andes in the 2000–2016 time period. Both contraction and expansion of croplands were detected in the Coast region with a 7% of overall net contraction. There was 8% increase in cropland in the highland páramo ecosystem. The Chocó rainforests were the most affected ecosystem in the Coast. Agricultural presence in the Amazon was lower than in the Coast and Andes, but still important, mainly in the North, and it is expanding rapidly. The remote sensing based estimates of land use change did not match well with the government statistics but were consistent with other studies. Our findings underline the need for the implementation of reliable sampling techniques and remote sensing methodologies to collect land cover data in order to understand how agriculture is affecting ecosystems in Ecuador.

## Supporting information

S1 Table Cropland area (km2) by province of Ecuador as estimated by us from Landsat data and as reported by the government of Ecuador for 2000 and 2016. * Santa Elena and Santo Domingo de los Tsáchilas provinces were created in 2007.
(DOCX)

S2 Table Cropland area (km2) by administrative-level region of Ecuador and for the entire country as estimated by us from Landsat data, and as reported by the government of Ecuador for 2000 and 2016. (DOCX)

S3 Table Cropland area (km2) by physiographic region as estimated by us from Landsat data, and as reported by the government of Ecuador for 2000 and 2016. It is important to highlight the census data is only available at administrative level, so this comparison is an approximation (Refer to Fig 1).
(DOCX)
