# Supplementary material for: Cropland expansion in Ecuador between 2000 and 2016
Source: PLoS One. 2023 Sep 19;18(9):e0291753. doi: 10.1371/journal.pone.0291753 (PMC10508625; doi:10.1371/journal.pone.0291753)
Supplement: S1 Table — * Santa Elena and Santo Domingo de los Tsáchilas provinces were created in 2007. (DOCX) [file pone.0291753.s001.docx]

**S1 Table.** Cropland area (km2) by province of Ecuador as estimated by us from Landsat data and as reported by the government of Ecuador for 2000 and 2016. * Santa Elena and Santo Domingo de los Tsáchilas provinces were created in 2007.

|  |  | **2000** |  | **2016** |  |
| --- | --- | --- | --- | --- | --- |
| **Province** | **code** | **Landsat** | **Government (census)** | **Landsat** | **Government (survey)** |
| Azuay | Azu | 2564 | 1457 | 2680 | 965 |
| Bolívar | Bol | 1317 | 2117 | 1415 | 1724 |
| Canar | Can | 1261 | 1041 | 1503 | 643 |
| Carchi | Car | 889 | 715 | 1133 | 519 |
| Cotopaxi | Cot | 2583 | 2072 | 2804 | 1276 |
| Chimborazo | Chi | 2238 | 1850 | 2962 | 1098 |
| Imbabura | Imb | 1270 | 1116 | 1462 | 572 |
| Loja | Loj | 931 | 2797 | 1000 | 1019 |
| Pichincha | Pic | 4671 | 5653 | 3460 | 1516 |
| Tungurahua | Tun | 1002 | 804 | 1415 | 569 |
| Santo D. T. | San | -- | -- | 1682 | 2017 |
| El Oro | El | 1584 | 3319 | 1211 | 1854 |
| Esmeraldas | Esm | 4531 | 4385 | 4093 | 4844 |
| Guayas | Gua | 8894 | 7021 | 8770 | 6752 |
| Los Ríos | Los | 5311 | 5437 | 5255 | 5261 |
| Manabí | Man | 5002 | 11877 | 4619 | 10992 |
| Santa Elena * | San | -- | -- | 370 | 189 |
| Morona Santiago | Mor | 140 | 4171 | 238 | 1496 |
| Napo | Nap | 21 | 1008 | 29 | 464 |
| Orellana | Ore | 559 | 796 | 835 | 509 |
| Pastaza | Pas | 77 | 2131 | 121 | 256 |
| Sucumbíos | Suc | 691 | 1304 | 1102 | 1624 |
| Zamora C. | Zam | 4 | 1010 | 10 | 869 |
| Total |  | 45540 | 62081 | 48169 | 47028 |
