# Supplementary material for: Cropland expansion in Ecuador between 2000 and 2016
Source: PLoS One. 2023 Sep 19;18(9):e0291753. doi: 10.1371/journal.pone.0291753 (PMC10508625; doi:10.1371/journal.pone.0291753)
Supplement: S2 Table — (DOCX) [file pone.0291753.s002.docx]

**S2 Table.** Cropland area (km2) by administrative-level region of Ecuador and for the entire country as estimated by us from Landsat data, and as reported by the government of Ecuador for 2000 and 2016

|  | **2000** | | **2016** | |
| --- | --- | --- | --- | --- |
| **Region** | **Landsat** | **Government**  **(census)** | **Landsat** | **Government**  **(survey)** |
| Coast | 18726 | 32039 | 21516 | 29892 |
| Sierra | 25322 | 19622 | 24318 | 11918 |
| Amazon | 1492 | 10420 | 2335 | 5218 |
