# Supplementary material for: Cropland expansion in Ecuador between 2000 and 2016
Source: PLoS One. 2023 Sep 19;18(9):e0291753. doi: 10.1371/journal.pone.0291753 (PMC10508625; doi:10.1371/journal.pone.0291753)
Supplement: S3 Table — It is important to highlight the census data is only available at administrative level, so this comparison is an approximation (Refer to Fig 1). (DOCX) [file pone.0291753.s003.docx]

**S3 Table.** Cropland area (km2) by physiographic region as estimated by us from Landsat data, and as reported by the government of Ecuador for 2000 and 2016. It is important to highlight the census data is only available at administrative level, so this comparison is an approximation (Refer to Figure 1)

|  | **2000** | | **2016** | |
| --- | --- | --- | --- | --- |
| **Physiographic region** | **Landsat** | **Government**  **(census)** | **Landsat** | **Government**  **(survey)** |
| Coast | 25322 | 32039 | 24318 | 29892 |
| Andes | 18724 | 19622 | 21516 | 11918 |
| Amazon | 1491 | 10420 | 2335 | 5218 |
